# The contribution of fiber components to water absorption of wheat grown in the UK

**DOI:** 10.1002/cche.10316

**Published:** 2020-07-16

**Authors:** Alison Lovegrove, Abigail J. Wood, Kirsty L. Hassall, Liz Howes, Mervin Poole, Paola Tosi, Peter Shewry

**Affiliations:** ^1^ Rothamsted Research Harpenden UK; ^2^ Heygates Ltd. Bugbrooke Mills Northampton UK; ^3^ School of Agriculture, Policy and Development University of Reading Whiteknights Campus Reading UK

**Keywords:** Farrand equation, fiber, water absorption, wheat

## Abstract

**Background and objectives:**

The water absorption (WA) of white wheat flour is a major factor affecting processing quality, and millers, therefore, process their wheat to achieve the required level. Although it is likely that WA is determined by the amounts and compositions of three major grain components, starch, protein, and arabinoxylan, the contribution of the latter is not agreed and not recognized in the widely used Farrand equation.

**Findings:**

We have measured a range of parameters related to fiber amount and composition and tested the ability of these to improve the prediction of WA using a modified Farrand equation. The addition of a range of single fiber traits improved the prediction of WA from a baseline of 82.98% to a maximum of 86.78%, but inclusion of all fiber traits as PCs resulted in a further improvement to 90%. Inclusion of the PCs also accounted for variation in WA between harvest years. The greatest improvement from inclusion of a single trait was observed with β‐glucan, the inclusion of arabinogalactan peptide (AGP) also resulted in improved prediction of WA.

**Conclusions:**

The study shows that fiber components contribute to variation in WA, including differences between harvest years, but that β‐glucan and AGP have similar or greater impacts than AX.

**Significance and novelty:**

The study dissects the contributions of AX amount and composition to WA and demonstrates a contribution of b‐glucan for the first time.

## 1 INTRODUCTION

The water absorption (WA) of white wheat flour, determined using either a Farinograph or DoughLAB (CCAT method no. 04), is a crucial test which is required to allow bakers to optimize the mixing conditions for baking. Hence, millers routinely adjust their milling conditions to achieve the level of WA agreed with customers.

Our current understanding of the contributions of grain components to WA was established in the 1950s.

Starch accounts for about 80% of white flour and hence is considered to have the greatest impact on WA. Intact granules absorb about 0.5‐times their dry weight of water, which increases to 3–4‐fold when damaged (Kent & Evers, 1994). Starch comprises two polymers, amylose and amylopectin, which generally account for about 30% and 70% of the total in wheat, respectively. Amylose and amylopectin have different WA capacity, with the swelling power of starch decreasing with increasing amylose content (Sasaki & Matsuki, 1998). Wheat starch granules show a bimodal size distribution, with large (10–40 μm) lenticular A granules and small (<10 μm) spherical B granules enriched in amylose and amylopectin, respectively. Higher temperatures during grain filling lead to an increase in the proportion of A granules, while water stress significantly decreases the number of B granules and reduces the size of the largest A granules (Brooks, Jenner, & Aspinall, 1982; Viswanathan & Khanna‐Chopra, 2001). The WA of starch will, therefore, be a function of the granule size distribution and granule composition, which are in turn affected by cultivar and environment.

Protein may affect WA due to differences in amount, composition and packing in the grain. Bread‐making flours all contain between 10.5% and 12.5% protein; therefore, variation in protein content is unlikely to be solely responsible for differences in WA. In general, proteins absorb approximately 1.8 times their dry weight of water, but this may vary between proteins and also be affected by their degree of polymerization, for example, the ratio of the gluten to soluble proteins (Rakszegi et al., 2014). Differences may also occur in the physical packing of the protein around the starch granules, giving rise to a vitreous or floury texture. Vitreousness is greater with high grain nitrogen and high growth temperatures (Kindred et al., 2008), but the physical chemical basis for this trait is not understood.

Finally, cell wall polysaccharides account for only 2%–3% of flour, with arabinoxylan (AX, often called pentosans) accounting for about 70% of this total (Mares & Stone, 1973). However, the total amount of AX varies from 1.35% to 2.75% (dry weight) between cultivars, and the water‐soluble fraction from 0.3% to 1.4% (dry weight) (Gebruers et al., 2008). Pentosans have a very high water‐holding capacity, about 10 times their dry weight for the water‐insoluble fraction and 11 times their dry weight for the water‐soluble faction (Finnie & Atwell, 2016; Guzmán, Posadas‐Romano, Hernández‐Espinosa, Morales‐Dorantes, & Peña, 2015). About 70% of the variation in total pentosans and 60% of the variation in soluble pentosans is determined by the cultivar, with smaller effects of the environment. In particular, the proportion of soluble AX is higher under cool wet conditions (Shewry et al., 2010).

Several early studies examined the prediction of WA based on protein and starch damage and concluded that pentosans did not contribute to the observed variation (Belderok, 1973; Dodds, 1972; Greer & Stewart, 1959; Tipples, Meredith, & Holas, 1978). This is reflected in the widely used Farrand equation (Farrand, 1969) which predicts WA based only on protein, starch damage, and moisture content:

$$\text{WA}=68.26+\left(0.878\times \%\text{protein}\right)+\left(0.334\times \text{damaged}\;\%\text{starch}\right)-\left(1.97\times \%\text{flour moisture}\right).$$

By contrast, Stevens and Stewart (1982) concluded that 14% of the 48% of the variation that was not accounted for by the Farrand equation could be explained by soluble pentosans. In a separate study, the addition of pentosanase (purified 1–4, β‐xylanase) had a negative impact on both dough rheology and baking quality (McCleary, Gibson, Allen, & Gams, 1986). The second major type of fiber in wheat flour is β‐glucan, which accounts for about 20% of the total cell wall polysaccharides (Mares & Stone, 1973). Β‐glucan has not been studied in detail from wheat flour but has been studied in barley and oats where it is the major cell wall polysaccharide and forms highly viscous solutions (Lazaridou & Biliaderis, 2007). Finally, white flour contains up to 0.4% dry weight of arabinogalactan peptide (AGP) (Loosveld, Grobet, & Delcour, 1997; Loosveld et al., 1998) which comprises a 15‐residue amino acid peptide (Van den Bulck et al., 2002) including three hydroxyprolines which are *o*‐glycosylated with branched arabinogalactan chains (Tryfona et al., 2010). AGP is not a cell wall component and most is extracted in water (Wilkinson et al., 2017). β‐glucan and AGP could also contribute to WA, and we therefore determined the amounts of both in this study.

For most grain samples, the dominant role of starch damage in determining WA means that millers can readily achieve the degree of WA required by bakers. However, over the last decade, the milling properties of all wheats (hard and soft) grown in the UK have varied widely between harvest years, with exceptionally high levels of energy being required in some years to achieve the WA required by bakers (resulting in higher costs and damage to the surface of rollers). We have therefore used a set of elite cultivars grown in years with typical (2018) and low (2017) WA to identify the components which contribute to this difference. Furthermore, to explore the specific role of pentosans, we have compared a series of lines with genetically determined differences in pentosan content of white flour.

## 2 MATERIALS AND METHODS

### 2.1 Field trials

Sixteen modern elite UK wheat cultivars were selected (Table S1). These included seven bread‐making cultivars of consistent quality (nabim group 1) and three of inconsistent quality (nabim group 2) released between 1987 and 2002, three cultivars suitable for biscuit‐making (nabim group 3) released between 1987 and 2003 and three cultivars suitable for livestock feed (nabim group 4). All were hard (expected SKCS units greater than 46) except the three biscuit wheats and one of the feed wheats. They were grown on the experimental farm at Rothamsted Research, Harpenden, UK in 2017 (all cultivars) and 2018 (all except Gallant) as part of the Wheat Genetic Improvement Network (WGIN) long‐term nitrogen use trials (Barraclough et al., 2010.), in triplicate randomized 9 × 3 m plots at 200 kgN/Ha (which is a typical nitrogen rate for the production of bread‐making wheat in the UK). Samples from the three replicate plots were milled separately as three biological replicates, except for Robigus grown in 2017, Cadenza in 2017, and Cordiale in both 2017 and 2018 where samples from only 2 plots were available.

Thirteen doubled haploid (DH) lines were selected from a cross between the cultivars Yumai 34 × Valoris. These two cultivars were used as parents because they have higher than average contents of arabinoxylan (AX) fiber (Lovegrove et al., 2020), and the lines were selected to display a range of AX contents. The thirteen lines and parents were grown at Rothamsted in 2018, in randomized 9 × 1.8 m plots with 200 kgN/ha.

### 2.2 Milling and determination of WA and starch damage

Wheat was dried to a maximum of 15% at a maximum of 30°C. Wheat was conditioned to 16% moisture with a lying time of 16–23 hr before milling. The wheat was milled at a rate of 100 g/min. Milling was carried out using a Brabender MLU 202 test mill and MLU 302 impact finisher according to established UK industry test milling requirements. WA was determined by Farinograph (CCAT method no.4) and the results presented on an “as is” basis. Starch damage was determined using the Chopin SDmatic (CCAT method no. 24) (which gives greater within‐laboratory precision than Farrand methods). Nitrogen was determined by Dumas combustion (ISO/TS 16634‐2:2009) and multiplied by 5.7 to give protein content.

### 2.3 Analysis of fiber components

Water‐extractable (WE)‐AX and total (TOT)‐AX were determined by monosaccharide analysis following mild acid hydrolysis as described by Bromley et al. (2013) with samples analyzed in triplicate. They are expressed as xylose units to avoid the requirement to correct arabinose values for arabinose present in AGP. However, this correction was made to estimate the ratio of arabinose to xylose in these fractions. The same monosaccharide analyses were used to determine AGP, which is expressed as galactose units to avoid the requirement to correct for arabinose present in AX.

TOT‐AX and β‐glucan were also determined as arabinoxylan oligosaccharides (AXOS) and gluco‐oligosaccharides (GOS), respectively, by enzyme fingerprinting as described by Freeman et al. (2017) with minor modifications. Flours were digested with recombinant endo‐1,4‐xylanase (PRO‐E0062) and lichenase (a glucan‐hydrolase) (PRO‐E0017) (Prozomix), the oligosaccharides separated by HPAEC, and the peak areas expressed relative to a Melibiose internal standard. Samples were analyzed in duplicate. This analysis also allowed the ratio of AX: β‐glucan to be calculated and provided information on β‐glucan structure (the ratio of GOS comprising three glucose (G3) and four glucose (G4) units released by digestion). The relative viscosity (RV) of water extracts was determined using the method of Freeman et al. (2016) using a Micro Ostwald capillary and WinVisco software (AVS 370; SI Analytics Germany).

### 2.4 Determination of grain hardness and vitreousness

Grain hardness was determined using the Perten Single Kernel Characterization System (SKCS) 4100 (Perten) on samples of 300 grains. Vitreousness was determined by visual examination and scoring and expressed as % vitreousness (ICC 129 Method, 1980). Grain was cut transversally using a single edge blade and placed in a 99‐well plate for visual scoring (33 kernels were cut for each plot); a total 7,722 kernels were scored.

### 2.5 Sucrose solvent retention capacity (SRC)

Solvent retention capacity (SRC) (Slade & Levine, 1994) determines the ability of flour to retain a set of solvents, including 50% sucrose which is preferentially absorbed by pentosans (and thus provides a measure of arabinoxylan) (Gaines, 2004). The sucrose SRC was therefore determined according to AACC Method 56‐11.02 (AACC International, 2009).

### 2.6 Proportion of propan‐1‐ol soluble gluten proteins

50% (v/v) propan‐1‐ol extracts gliadins and a proportion of glutenin polymers (low molecular weight polymers enriched in LMW subunits if glutenin) from wheat flour, whereas high molecular weight glutenin polymers are extracted using the same solvent with a reducing agent. The proportions of these fractions (called gliadins and glutenins, respectively, for convenience) were therefore determined by sequential extraction with the 50% (v/v) propan‐1‐ol without and with a reducing agent. 100 mg aliquots of flour of each sample were extracted, independently, with 2 ml 50% (v/v) propan‐1‐ol or 2 ml 50% (v/v) propan‐1‐ol, 25 mM Tris pH 6,8, 2% dithiothreitol (DTT). Extraction was carried out at room temperature for 20 min with constant shaking, following which samples were centrifuged at 6,500 *g* for 10 min. The protein in the supernatant was quantified using the Direct Detect^®^ Infrared Spectrometer (Merk) following the manufacturers’ instructions.

### 2.7 Statistical analysis/modeling

For the WGIN samples, linear models were fitted to explain variation in water absorption (response). A baseline model was defined to include all variables of the Farrand equation (starch damage, protein and flour moisture). This baseline model was compared to models including these baseline variables and a single dietary fiber component (of which there were 15), and to a model incorporating all 15 dietary fiber components. To avoid issues of collinearity, the 15 dietary fiber components were first processed through a principal component analysis based on the correlation matrix, resulting in up to 10 principal components (PCs), accounting for more than 99% of the variation, to be included in the linear model. A reduced all‐fiber model was defined by sequentially omitting nonsignificant PCs from the model as assessed at the 5% significance level. Models were then compared using the adjusted R^2^, a measure of percentage variance in the response, WA, that is accounted for by the model. A second set of models was fitted by including an additional factor associated with the year sampled. The aim here was to investigate whether the fiber trait accounted for the variation observed between years and, as such, involved an assessment of whether year had a significant effect in the model (by consideration of the marginal *F*‐statistic) and whether the adjusted *R* ^2^ improved by including year.

For the Yumai 34 × Valoris samples, models were fitted in the same process as above. Given the smaller sample size, only seven PCs, accounting for 99% of the variation, were considered for the full fiber model.

## 3 RESULTS

### 3.1 Comparison of elite cultivars grown in years with typical (2018) and low (2017) WA

Sixteen cultivars were selected, all of which have been grown commercially in the UK over the past 25 years (Table S1). They represented all types of wheat grown in the UK: hard bread‐making wheats (nabim groups 1 and 2), soft biscuit wheats (nabim group 3), and both hard and soft feed wheats (nabim group 4). Samples were harvested from field trials from two years which differed in the level of WA achieved by millers: 2018 which was considered by the UK industry to be typical and 2017 which was considered to be unusually low (“atypical”).

**FIGURE 1:**
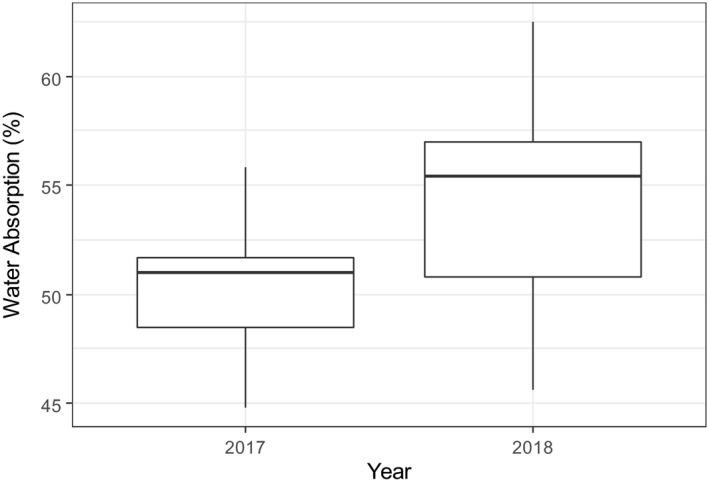
Boxplots for water absorption of flours from samples grown in 2017 (low WA) and 2018 (typical WA). Boxes delineate the upper and lower quartiles. Whiskers represent upper and lower values, and means are represented by a solid line within boxes

Replicate samples from each year were milled and the WA determined using industry standard methods. The mean WA was statistically different between years (*F*_1,58_ = 99.76, *p* < .001) and also between varieties (*F*_16,58_ = 16.01, *p* < .001); however, there was little evidence of an interaction between the two (*F*_13,58_ = 1.78, *p* = .068). Clearly, WA was lower in 2017 than in 2018 (Figure 1), confirming that the year was “atypical,” but the range of WAs for the two years overlapped.

The flours were analyzed for a range of components and properties, selected based on their potential to influence water absorption: starch damage, protein content, moisture content, percentage gliadin, hardness, vitreousness, and a number of parameters specifically related to fiber content and composition (including amounts and properties of AX, β‐glucan and AGP, RV, and sucrose SRC). These parameters are summarized in Table S2.

### 3.2 Correlations and multivariate analysis of parameters measured in grain samples grown in the two years

The Farrand equation (Farrand, 1969) was used as the basis for exploring the effects of grain components on WA, using regression analysis. To avoid confounding effects in the analysis, correlations between the individual parameters were initially determined and are shown as heat maps in Figures S1 and S2. These show strong correlations between % starch damage, hardness, and vitreousness meaning that only one of these measurements needed be included as an explanatory variable. Similarly, % gliadin was correlated with % protein and only one of these parameters needed to be included. To be consistent with the Farrand equation, % starch damage and % protein were chosen.

**FIGURE 2:**
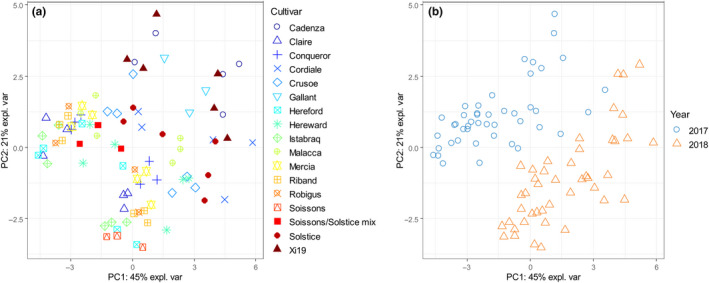
PCA (PC1 v PC2) of dietary fiber components, by cultivar (a) and years (b) [Colour figure can be viewed at wileyonlinelibrary.com]

Correlations are also observed between fiber components, notable water‐extractable WE‐AX (expressed as WE‐X) and the relative viscosity of aqueous extracts (Figure S2). Rather than selecting a subset of independent fiber measurements for further analysis, we combined the fiber data by principal component analysis (PCA). This identified 10 principal components which together accounted for 99% of the variation in the dataset. Figure 2 shows plots of PC1 against PC2, which together account for 67% of the total variance, colored to show the cultivars (A) and years (B). This shows a clear separation between years. The contributions of the traits to the PCs (loadings) are given in Table S3.

### 3.3 Modeling the contributions of fiber components to WA

In order to determine which components are of most importance in determining WA, we developed a statistical model based upon the analytical data. A baseline regression model (based on the Farrand equation) was defined by:

WA=%Starch damage+%Protein+%Flour Moisture.

This model accounted for 83% of the variation in water absorption (adjusted *R* ^2^). The model was then modified by introducing either individual fiber traits or the PCs discussed above. The full results are shown in Table S4 and are summarized in Table 1 (% variance explained without year column). Tables S8 and S9 provide mean, min and max for all samples in all years.

**TABLE 1:** Regression analysis of WA, adding fiber components to the baseline equation

Model	Additional traits or PCs	% variance explained without year	% variance explained with year
Baseline	None	82.98	87.91
Fiber 1	Relative viscosity	**83.01**	**88.23**
Fiber 2	WE‐AX (measured as X)	82.85	**88.00**
Fiber 3	Ratio of A:X in WE‐AX	**83.76**	87.91
Fiber 4	Sucrose SRC	**84.07**	**88.51**
Fiber 5	Total A (from AX and AGP)	**84.86**	87.79
Fiber 6	Ratio of A:X in TOT‐AX	**85.46**	87.77
Fiber 7	TOT‐AX (measured as X)	**83.19**	87.77
Fiber 8	Ratio of WE‐AX:TOT‐AX (measured as X)	**83.67**	**88.13**
Fiber 9	Total β‐glucan	**86.78**	**90.29**
Fiber 10	Ratio of G3:G4 oligosaccharides in β‐glucan	82.82	87.82
Fiber 11	Ratio of AX: β‐glucan (measured by fingerprinting)	82.79	**88.73**
Fiber 12	WE‐galactose (from AGP)	**85.12**	87.79
Fiber 13	TOT‐galactose (from AGP)	**85.31**	87.77
Fiber 14	TOT‐AX (measured by fingerprinting)	**84.85**	87.83
Fiber 15	WE‐A (from AX and AGP)	**84.60**	**88.36**
All PCs	PCs1 to 10	**89.88**	**89.79**
Reduced fiber PCs	PC1 + 2+4 + 5+6 + 9	**90.18**	**90.06**

Note

The % variance is calculated with and without including year. Values above the baseline are shown in bold.

Small improvements were obtained by including individual fiber traits, including the relative viscosity of aqueous extracts (RV) and parameters relating to the amount, structure, and solubility of AX. However, improvements also occurred when including galactose (WE and TOT) which is mainly derived from AGP, and the greatest improvement observed by including a single trait was with β‐glucan not AX. The greatest improvement overall was observed when the PCs were included, either all 10 PCs (to 89.9%) or only six (1, 2, 4, 5, 6 ,9) (to 90.2%). These 6 PCs account for 84.28% of the variation (Table S2), compared with 99% for all ten PCs.

### 3.4 Modeling the contributions of components to differences in WA between years

It is clear from Figure 1 that WA differs between years, and to determine whether the improved models could also explain this variation, we compared each of the models in Table 1 to a corresponding model that additionally includes year as a factor. The full details are given in Table S5 and the results summarized in Table 1 (% variance with year column). Without exception, addition of the individual traits failed to explain the variation in WA over years (year remained a significant factor in the models). Furthermore, the effect of year was greater than the effect of the individual fiber trait (marginal *F*‐statistic associated with year is bigger than the marginal *F*‐statistic associated with the trait, Table S5).

However, when all of the fiber traits were considered by including the PCs in the regression, differences due to year were no longer apparent (with nonsignificant effects, see Tables S4 and S5) and similar adjusted *R* ^2^ values were obtained: of 89.88 and 89.79 (including and excluding year, respectively) for the models with all PCs and of 90.18 and 90.06 (including and excluding year, respectively) and with six PCs only.

This shows that differences in fiber content and composition were responsible for the differences in WA between the years, but that it could not be ascribed to a single fiber trait (it was necessary to include the PCs based on all fiber traits in the model).

### 3.5 Comparison of lines with different contents of AX

**FIGURE 3:**
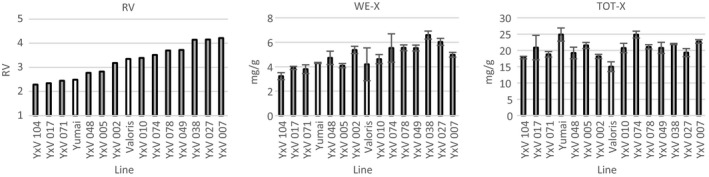
Relative viscosity (RV) and contents of WE‐X and TOT‐X in 13 DH lines (solid columns) from the cross Yumai 34 × Valoris and the two parental lines (open columns) [Colour figure can be viewed at

To explore the effects of the major fiber component, AX (pentosan), on WA in more detail, we compared a series of doubled haploid lines selected from a cross between two parents with unusually high contents of AX, Yumai 34, and Valoris (Lovegrove et al., 2020). The lines were selected to exhibit variation in AX content in a randomly segregating genetic background and grown with the parents in randomized field plots. The RV and contents of WE‐X and TOT‐X of white flours from the lines are shown in Figure 3.

**FIGURE 4:**
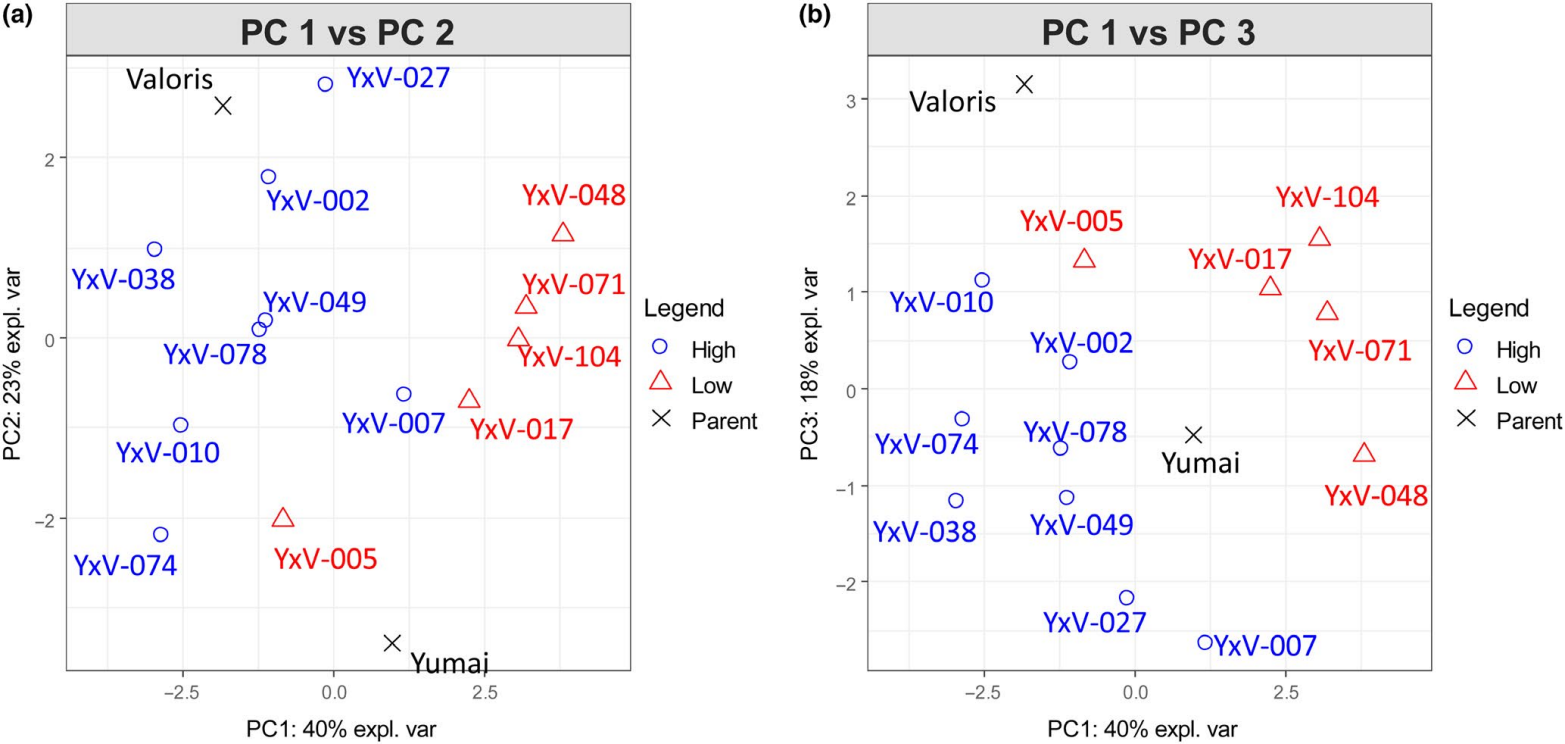
The first three principal axes from PCA of fiber components in the 13 DH lines differing in fiber content high (blue circles), low (red triangles), and the parental lines (black crosses). (a) PC‐1 vs PC‐2; (b) PC‐1 vs PC‐3 [Colour figure can be viewed at wileyonlinelibrary.com]

The same range of parameters was measured as for the WGIN samples above and the fiber traits analyzed by PCA. This identified seven PCs which together accounted for 99% of the total variance (Table S5). The first three PCs together accounted for 81% of the total variance, and plots of PC1 against PC2 and PC3 are shown in Figure 4. Both show good separation of the samples, with the parental lines clearly separated from the progeny. The loadings for all seven PCs are given in Table S6.

Regression analysis was carried out as above, using the baseline model based on the Farrand equation which was then modified by introducing either individual fiber traits or the PCs. The baseline model gave an approximate percentage variance accounting for 86.01% of the variation, so only traits which improved on this value are listed in Table S7 and summarized in Table 2.

**TABLE 2:** Summary of regression analysis of WA of flours from the Yumai 34 × Valoris lines and parents, adding fiber components to the baseline equation

Model	Trait or PC added	% variance accounted for
Baseline	None	86.01
Fiber 1	RV	94.21
Fiber 2	WE‐AX (measured as X)	96.73
Fiber 3	Ratio of A:X in TOT‐AX	89.10
Fiber 4	TOT‐AX (measured as X)	87.61
Fiber 5	Ratio of WE‐AX:TOT‐AX (measured as X)	90.64
Fiber 6	Total β‐glucan	91.33
Fiber all PC	PC1 + 2+3 + 4+5 + 6+7	95.18
Fiber PC1 + 3	PC1 + PC3	96.77

The addition of RV improved the prediction to 94.21% while the addition of several traits related to AX amount and composition also gave improvements, to 90.64%, for example, for the ratio of WE:TOT‐X. However, the greatest improvement achieved by adding a single fiber component was obtained with addition of total β‐glucan, to 91.33%.

The best improvement was achieved by adding PCs 1 and 3 which account for 57.69% of the variance in the dataset (Table S7). Adding these two PCs to the baseline model improved the variance accounted for to 96.77% (Table 2). A smaller improvement also resulted from including all fiber PCs, to 95.18.

However, addition of all fiber PCs, RV, and WE‐X also gave improvements, to 95.18%, 94.21%, and 96.73%, respectively.

## 4 DISCUSSION

Water absorption is one of the major determinants of the end‐use quality of white flour, particularly for bread making, but also for other food manufacturing processes. There is no doubt that the three major factors determining WA are grain protein content, starch damage, and grain moisture, and these are the basis of the widely used Farrand equation. In the present study, these three parameters predicted between 83% and 86% of the variation in WA in flours from UK cultivars and genetic lines but failed to account for the year‐to‐year variation in WA which is of concern to millers and bakers. Fiber components are also known to have high water‐binding capacity, and the contribution of AX to WA has been noted by several authors (e.g., Izydorczyk & Biliaderis, 1992; Tipples et al., 1978; Wang, Oudgenoeg, van Vliet, & Hamer, 2003), and we therefore measured a number of parameters relating to their amount, composition, and properties and determined the ability of these to improve the prediction of WA using a modified Farrand equation. The addition of individual parameters contributed small improvements to the prediction of WA in a set of cultivars grown in 2017 and 2018, from 82.98% up to 86.78% and when the fiber traits were combined using PCs the predictions increased to about 90%. In order to determine whether these parameters also accounted for the differences between years, the analysis was carried out including year as a factor. This improved the baseline prediction to almost 88%, but the addition of single traits resulted in little or no improvement. By contrast, when the PCs were included, the prediction was increased to 90%, which was similar to that when year was not included as a factor. This demonstrates that variation in fiber components accounted for the difference between years, but that it could not be ascribed to any single component.

AX is the major fiber component in white flour, accounting for 1.35%–2.7% of white flour (Gebruers et al., 2008). It was therefore expected to have the greatest impact on WA (Biliaderis, Izydorczyk, & Rattan, 1995; Rakha, Amana, & Andersson, 2013; Roels, Cleemput, Vandewalle, & Delcour, 1993). However, we found that β‐glucan and AGP made greater single contributions to improving the prediction of WA in both soft and hard wheats than most traits related to AX amount and composition. These components account for about 0.5% or less of white flour and neither has been studied in relation to effects on milling and WA.

β‐glucan also made a greater contribution than total AX to the variation in WA in a second set of lines, derived from a cross between the cultivars Yumai 34 and Valoris. In this case, WE‐AX had the greatest single effect on WA, as might be expected, as these lines were selected to exhibit variation in soluble AX content in a segregating background but had not been analyzed previously for variation in β‐glucan content. No contribution of AGP to variation in WA was observed in this population, with little variation in amount being observed between the lines.

## 5 CONCLUSIONS

Hence, our study shows that the prediction of WA by the Farrand equation can be improved by including fiber components, but that it is necessary to consider the contributions of β‐glucan and AGP as well as the major AX (pentosan) components.

## Supporting information

Data S1

Data S2

Data S3
