# Supplementary material for: The contribution of fiber components to water absorption of wheat grown in the UK
Source: Cereal Chem. 2020 Jul 16;97(5):940–8. doi: 10.1002/cche.10316 (PMC7540380; doi:10.1002/cche.10316)
Supplement: Supplementary file 1 — Data S1 [file CCHE-97-940-s001.docx]

Lovegrove A, Wood AJ, Kirsty L Hassall KL, Howes E, Mervin Poole M, Tosi P, Shewry PR

**Table S1.** WGIN cultivars used for analysis with nabim group, grain texture and year of release**.**

| Cultivar | nabim group | Grain texture | Year of Release |
| --- | --- | --- | --- |
| Mercia | 1 | Hard | 1985 |
| Hereward | 1 | Hard | 1989 |
| Malacca | 1 | Hard | 1997 |
| Solstice | 1 | Hard | 2002 |
| Xi19 | 1 | Hard | 2002 |
| Gallant | 1 | Hard | 2009 |
| Crusoe | 1 | Hard | 2012 |
| Soissons | 2 | Hard | 1987 |
| Cadenza | 2 | Hard | 1992 |
| Cordiale | 2 | Hard | 2000 |
| Riband | 3 | Soft | 1987 |
| Claire | 3 | Soft | 1999 |
| Robigus | 3 | Soft | 2003 |
| Istabraq | 4 | Soft | 2004 |
| Hereford | 4 | Hard | 2007 |
| Conqueror | 4 | Hard | 2009 |

**Supplementary Table S2.** Traits measured in all flour samples.

| Type of trait | Trait | Analytical method | Shorthand in Tables and Figures |
| --- | --- | --- | --- |
| Starch | % Starch damage (NIR) | NIR | Starch damage |
| Protein | % Protein | Dumas combustion of flour protein (Nx5.7) ‘as is’ in 2017 and 2018 samples | Protein |
|  | Gliadin as % total gluten protein | Direct Detect® Infrared Spectrometer | % gliadin |
| Water | % Flour moisture | NIR | Flour moisture |
| Texture | Hardness | SKCS-Perten | Hardness |
|  | % Vitreousness | Visual scoring from sectioning | Vitreousness |
| Fibre | Water extractable arabinose: from AX and AGP | Monosaccharide analysis | WE-A |
|  | Water-extractable arabinoxylan (WE-AX) | Monosaccharide analysis, expressed as xylose | WE-X |
|  | Arabinose:xylose ratio in WE-AX | Monosaccharide analysis | WE-A:X |
|  | Relative viscosity | Capillary viscometry of aqueous extracts | RV |
|  | Sucrose solvent retention capacity | ACCI Method 56-11.02 | Sucrose SRC |
|  | Total arabinose in AX | Monosaccharide analysis expressed, as arabinose (adjusted for AGP) | TOT-A |
|  | Total arabinoxylan (AX) | Monosaccharide analysis, expressed as xylose | TOT-X |
|  | Arabinose:xylose ratio in TOT-AX | Monosaccharide analysis | TOT-A:X |
|  | AX (without xylose) | Enzymatic fingerprinting | TOT-AX (-X1) |
|  | Ratio of WE-AX:TOT-AX | Monosaccharide analysis, expressed as xylose | WE:TOT X |
|  | Total β-glucan (BG) | Enzymatic fingerprinting expressed in arbitrary units | TOT-BG |
|  | β-glucan structure | Ratio of G3:G4 gluco-oligosaccharides released enzymatic fingerprinting of β-glucan | G3:G4 BG |
|  | Ratio of AX: β-glucan | Ratio of AX: β-glucan from enzymatic fingerprinting | AX:BG |
|  | Soluble arabinogalactan peptide (AGP) | WE- AGP from monosaccharide analysis, expressed as galactose | WE-G |
|  | Total arabinogalactan peptide (AGP) | TOT-AGP from monosaccharide analysis, expressed as galactose | TOT-G |

**Table S3.** Contributions (loadings) of fibre components to PCs 1 to 10.

| **Percentage variance of each PC** | 45.28% | 21.26% | 11.69% | 6.68% | 5.96% | 4.13% | 1.90% | 1.15% | 0.97% | 0.48% |
| --- | --- | --- | --- | --- | --- | --- | --- | --- | --- | --- |
|  |  |  |  |  |  |  |  |  |  |  |
|  | **PC1** | **PC2** | **PC3** | **PC4** | **PC5** | **PC6** | **PC7** | **PC8** | **PC9** | **PC10** |
| **RV** | 0.330605 | 0.013849 | -0.22261 | 0.254341 | -0.07071 | 0.276023 | 0.25628 | 0.006267 | 0.025389 | -0.07553 |
| **WE-A** | 0.352924 | -0.13165 | -0.17006 | 0.07058 | 0.044694 | 0.132941 | -0.21824 | 0.132403 | -0.23989 | -0.18304 |
| **WE-X** | 0.362348 | 0.034175 | -0.17456 | 0.020044 | -0.14193 | 0.152693 | -0.0561 | -0.06063 | -0.28101 | 0.007199 |
| **WE-A:X** | -0.28265 | -0.22571 | -0.14429 | 0.265735 | 0.271975 | 0.224113 | 0.124334 | 0.649899 | -0.18255 | -0.31003 |
| **Sucrose SRC** | 0.345003 | -0.08366 | 0.004875 | 0.185162 | 0.041954 | 0.171381 | 0.274706 | 0.015323 | 0.764488 | -0.06781 |
| **TOT-A** | 0.061338 | 0.499036 | 0.061825 | 0.151683 | 0.244085 | 0.340807 | -0.24038 | -0.1299 | -0.06643 | 0.014524 |
| **TOT-X** | 0.295342 | 0.283791 | 0.172099 | 0.170361 | 0.211271 | 0.011584 | 0.106608 | -0.21548 | -0.29594 | -0.19663 |
| **TOT-A:X** | -0.26632 | 0.304909 | -0.11351 | 0.002255 | 0.044302 | 0.439564 | -0.44175 | 0.144816 | 0.280462 | 0.226012 |
| **TOT-AX (-X1)** | 0.194268 | 0.436421 | 0.038616 | -0.16707 | -0.16109 | -0.26079 | 0.080064 | 0.457518 | 0.027996 | -0.03542 |
| **WE:TOT-X** | 0.221591 | -0.23293 | -0.40786 | -0.13743 | -0.40187 | 0.117315 | -0.25045 | 0.091071 | -0.06339 | 0.290903 |
| **TOT-BG** | 0.15563 | 0.095427 | 0.537626 | -0.3646 | -0.31405 | 0.286944 | 0.108672 | 0.361362 | -0.04902 | -0.03071 |
| **G3:G4 BG** | 0.061467 | -0.0002 | -0.31307 | -0.73241 | 0.497047 | 0.192742 | 0.267652 | -0.068 | -0.00722 | 0.004574 |
| **AX:BG** | 0.07932 | 0.39489 | -0.40755 | 0.069886 | 0.083849 | -0.48076 | 0.014486 | 0.249782 | 0.098444 | 0.074716 |
| **WE-G** | 0.285241 | -0.22006 | 0.162598 | -0.16485 | 0.25406 | -0.22979 | -0.6128 | 0.070036 | 0.220564 | -0.39357 |
| **TOT-G** | 0.26743 | -0.20646 | 0.276501 | 0.15743 | 0.43156 | -0.09237 | -0.00439 | 0.2275 | -0.09395 | 0.723007 |

**Table S4** Regression analysis of WA, adding fibre components to the Baseline equation

| **Model** | **Term** | **Mean square** | **F value** | **P value** | **% variance explained** |
| --- | --- | --- | --- | --- | --- |
| Baseline | Starch damage | 313.671 | 116.7183 | < 2.2e-16 | 82.98952 |
|  | Protein | 0.852 | 0.317 | 0.574895 |  |
|  | Flour Moisture | 22.434 | 8.3477 | 0.004899 |  |
| fibre 1 | Starch damage | 299.823 | 111.7429 | < 2.2e-16 | 83.01656 |
|  | Protein | 1.753 | 0.6533 | 0.421234 |  |
|  | Flour Moisture | 23.262 | 8.6697 | 0.004184 |  |
|  | Relative viscosity | 3.046 | 1.1353 | 0.289692 |  |
| fibre 2 | Starch damage | 286.09 | 105.6161 | < 2.2e-16 | 82.85435 |
|  | Protein | 1.329 | 0.4907 | 0.485561 |  |
|  | Flour Moisture | 22.259 | 8.2175 | 0.005243 |  |
|  | WE-X | 0.894 | 0.3299 | 0.567273 |  |
| fibre 3 | Starch damage | 180.631 | 70.4179 | 9.95E-13 | 83.76354 |
|  | Protein | 0.802 | 0.3127 | 0.57751 |  |
|  | Flour Moisture | 30.925 | 12.0558 | 0.000818 |  |
|  | WE-A:X | 12.959 | 5.0521 | 0.027216 |  |
| fibre 4 | Starch damage | 288.061 | 114.4834 | < 2.2e-16 | 84.07339 |
|  | Protein | 8.688 | 3.4528 | 0.066646 |  |
|  | Flour Moisture | 28.402 | 11.2876 | 0.001175 |  |
|  | Sucrose SRC | 17.071 | 6.7846 | 0.010872 |  |
| fibre 5 | Starch damage | 247.812 | 103.5717 | 2.57E-16 | 84.85525 |
|  | Protein | 5.376 | 2.2467 | 0.137647 |  |
|  | Flour Moisture | 22.283 | 9.3132 | 0.003045 |  |
|  | TOT-A | 27.447 | 11.4714 | 0.001077 |  |
| fibre 6 | Starch damage | 345.44 | 150.4271 | < 2.2e-16 | 85.46466 |
|  | Protein | 8.85 | 3.8545 | 0.05292 |  |
|  | Flour Moisture | 26.58 | 11.5733 | 0.001027 |  |
|  | TOT-A:X | 35.53 | 15.4741 | 0.000172 |  |
| fibre 7 | Starch damage | 216.616 | 81.5633 | 5.13E-14 | 83.18967 |
|  | Protein | 1.167 | 0.4393 | 0.509255 |  |
|  | Flour Moisture | 20.46 | 7.7039 | 0.006791 |  |
|  | TOT-X | 5.344 | 2.012 | 0.159757 |  |
| fibre 8 | Starch damage | 312.66 | 121.2011 | < 2.2e-16 | 83.6715 |
|  | Protein | 4.395 | 1.7038 | 0.19535 |  |
|  | Flour Moisture | 18.343 | 7.1104 | 0.009191 |  |
|  | WE:TOT-X | 11.738 | 4.5501 | 0.035834 |  |
| fibre 9 | Starch damage | 209.296 | 100.227 | 5.51E-16 | 86.78225 |
|  | Protein | 0.693 | 0.332 | 0.566026 |  |
|  | Flour Moisture | 25.616 | 12.267 | 0.000741 |  |
|  | TOT-BG | 53.02 | 25.39 | 2.65E-06 |  |
| fibre 10 | Starch damage | 312.719 | 115.1116 | < 2.2e-16 | 82.80442 |
|  | Protein | 0.804 | 0.296 | 0.587858 |  |
|  | Flour Moisture | 22.411 | 8.2494 | 0.005159 |  |
|  | G3:G4 BG | 0.231 | 0.085 | 0.771301 |  |
| fibre 11 | Starch damage | 293.983 | 108.1499 | < 2e-16 | 82.79412 |
|  | Protein | 0.945 | 0.3477 | 0.55697 |  |
|  | Flour Moisture | 22.445 | 8.2571 | 0.00514 |  |
|  | AX:BG | 0.094 | 0.0347 | 0.85273 |  |
| fibre 12 | Starch damage | 339.5 | 144.4656 | < 2.2e-16 | 85.12508 |
|  | Protein | 8.82 | 3.7517 | 0.056116 |  |
|  | Flour Moisture | 28.24 | 12.016 | 0.000834 |  |
|  | WE-G | 31.03 | 13.2032 | 0.00048 |  |
| fibre 13 | Starch damage | 334.23 | 144.0926 | < 2.2e-16 | 85.31811 |
|  | Protein | 10.55 | 4.5465 | 0.035907 |  |
|  | Flour Moisture | 40.56 | 17.4845 | 7.08E-05 |  |
|  | TOT-G | 33.59 | 14.4813 | 0.000268 |  |
| fibre 14 | Starch damage | 174.194 | 72.771 | 5.23E-13 | 84.84845 |
|  | Protein | 6.081 | 2.5403 | 0.114727 |  |
|  | Flour Moisture | 24.497 | 10.2337 | 0.001946 |  |
|  | TOT-AX (-X1) | 27.357 | 11.4286 | 0.001099 |  |
| fibre 15 | Starch damage | 336.98 | 138.4849 | < 2.2e-16 | 84.59775 |
|  | Protein | 8.69 | 3.5696 | 0.062297 |  |
|  | Flour Moisture | 28.46 | 11.6944 | 0.00097 |  |
|  | WE-A | 24.03 | 9.8753 | 0.002315 |  |
| fibre all PC | Starch damage | 143.582 | 89.8464 | 1.84E-14 | 89.88465 |
|  | Protein | 22.142 | 13.8551 | 0.00038 |  |
|  | Flour Moisture | 34.316 | 21.4732 | 1.48E-05 |  |
|  | fibre_PC_1 | 11.379 | 7.1203 | 0.009336 |  |
|  | fibre_PC_2 | 55.054 | 34.4499 | 1.11E-07 |  |
|  | fibre_PC_3 | 2.393 | 1.4973 | 0.224922 |  |
|  | fibre_PC_4 | 14.701 | 9.199 | 0.003325 |  |
|  | fibre_PC_5 | 16.514 | 10.3336 | 0.001928 |  |
|  | fibre_PC_6 | 7.101 | 4.4435 | 0.038376 |  |
|  | fibre_PC_7 | 0.514 | 0.3214 | 0.572437 |  |
|  | fibre_PC_8 | 0.05 | 0.0311 | 0.860423 |  |
|  | fibre_PC_9 | 2.724 | 1.7043 | 0.195714 |  |
|  | fibre_PC_10 | 0.116 | 0.0726 | 0.788318 |  |
| fibre reduced PC | Starch damage | 209.394 | 135.0301 | < 2.2e-16 | 90.18445 |
|  | Protein | 27.417 | 17.6799 | 6.84E-05 |  |
|  | Flour Moisture | 46.441 | 29.9479 | 5.07E-07 |  |
|  | fibre_PC_1 | 24.405 | 15.7378 | 0.000159 |  |
|  | fibre_PC_2 | 63.872 | 41.1888 | 9.47E-09 |  |
|  | fibre_PC_4 | 16.057 | 10.3547 | 0.001874 |  |
|  | fibre_PC_5 | 17.946 | 11.5725 | 0.001053 |  |
|  | fibre_PC_6 | 6.334 | 4.0847 | 0.04666 |  |
|  | fibre_PC_9 | 5.044 | 3.2529 | 0.075108 |  |

**Table S5:** Regression analysis incorporating Year

| **Model** | **Term** | **Mean square** | **F value** | **P value** | **% variance explained** | **% variance explained (without Year)** |
| --- | --- | --- | --- | --- | --- | --- |
| Baseline | Starch damage | 325.16 | 170.25 | < 2.2e-16 | 87.91097 | 82.98952 |
|  | Protein | 20.71 | 10.845 | 0.001451 |  |  |
|  | Flour Moisture | 55.6 | 29.111 | 6.20E-07 |  |  |
|  | Year | 68 | 35.603 | 5.56E-08 |  |  |
| fibre 1 | Starch damage | 318.33 | 171.2244 | < 2.2e-16 | 88.23222 | 83.01656 |
|  | Protein | 25.35 | 13.637 | 0.000396 |  |  |
|  | Flour Moisture | 58.29 | 31.3518 | 2.72E-07 |  |  |
|  | Relative viscosity | 6.12 | 3.2931 | 0.073183 |  |  |
|  | Year | 71.08 | 38.2301 | 2.26E-08 |  |  |
| fibre 2 | Starch damage | 305.344 | 161.0303 | < 2.2e-16 | 87.99774 | 82.85435 |
|  | Protein | 23.653 | 12.4742 | 0.000677 |  |  |
|  | Flour Moisture | 56.061 | 29.565 | 5.33E-07 |  |  |
|  | WE-X | 3.048 | 1.6073 | 0.208416 |  |  |
|  | Year | 70.153 | 36.997 | 3.48E-08 |  |  |
| fibre 3 | Starch damage | 212.229 | 111.153 | < 2.2e-16 | 87.91452 | 83.76354 |
|  | Protein | 18.68 | 9.7836 | 0.002429 |  |  |
|  | Flour Moisture | 57.552 | 30.1425 | 4.28E-07 |  |  |
|  | WE-A:X | 1.956 | 1.0247 | 0.31435 |  |  |
|  | Year | 56.996 | 29.8513 | 4.78E-07 |  |  |
| fibre 4 | Starch damage | 278.301 | 153.399 | < 2.2e-16 | 88.51653 | 84.07339 |
|  | Protein | 29.936 | 16.5005 | 0.00011 |  |  |
|  | Flour Moisture | 59.909 | 33.0217 | 1.46E-07 |  |  |
|  | Sucrose SRC | 9.851 | 5.4296 | 0.022226 |  |  |
|  | Year | 60.778 | 33.5009 | 1.23E-07 |  |  |
| fibre 5 | Starch damage | 281.439 | 145.95 | < 2.2e-16 | 87.79435 | 84.85525 |
|  | Protein | 20.669 | 10.7184 | 0.001548 |  |  |
|  | Flour Moisture | 49.75 | 25.7998 | 2.29E-06 |  |  |
|  | TOT-A | 0.381 | 0.1974 | 0.657973 |  |  |
|  | Year | 40.933 | 21.2271 | 1.46E-05 |  |  |
| fibre 6 | Starch damage | 306.551 | 158.745 | < 2.2e-16 | 87.77681 | 85.46466 |
|  | Protein | 20.815 | 10.779 | 0.001504 |  |  |
|  | Flour Moisture | 50.698 | 26.253 | 1.92E-06 |  |  |
|  | TOT-A:X | 0.151 | 0.078 | 0.780662 |  |  |
|  | Year | 32.615 | 16.89 | 9.26E-05 |  |  |
| fibre 7 | Starch damage | 248.261 | 128.4938 | < 2.2e-16 | 87.77055 | 83.18967 |
|  | Protein | 20.53 | 10.6258 | 0.001618 |  |  |
|  | Flour Moisture | 53.238 | 27.5545 | 1.15E-06 |  |  |
|  | TOT-X | 0.069 | 0.0355 | 0.85099 |  |  |
|  | Year | 62.724 | 32.4646 | 1.80E-07 |  |  |
| fibre 8 | Starch damage | 323.94 | 172.7885 | < 2.2e-16 | 88.13322 | 83.6715 |
|  | Protein | 24.74 | 13.1981 | 0.000484 |  |  |
|  | Flour Moisture | 48.64 | 25.943 | 2.17E-06 |  |  |
|  | WE:TOT-X | 4.82 | 2.5732 | 0.112483 |  |  |
|  | Year | 61.09 | 32.5827 | 1.72E-07 |  |  |
| fibre 9 | Starch damage | 228.253 | 148.799 | < 2.2e-16 | 90.29045 | 86.78225 |
|  | Protein | 15.497 | 10.102 | 0.002081 |  |  |
|  | Flour Moisture | 52.797 | 34.418 | 8.79E-08 |  |  |
|  | TOT-BG | 33.112 | 21.585 | 1.26E-05 |  |  |
|  | Year | 48.091 | 31.35 | 2.72E-07 |  |  |
| fibre 10 | Starch damage | 325.17 | 168.9307 | < 2.2e-16 | 87.81619 | 82.80442 |
|  | Protein | 20.49 | 10.6437 | 0.001604 |  |  |
|  | Flour Moisture | 55.72 | 28.9483 | 6.74E-07 |  |  |
|  | G3:G4 BG | 0.67 | 0.3466 | 0.557662 |  |  |
|  | Year | 68.44 | 35.5531 | 5.83E-08 |  |  |
| fibre 11 | Starch damage | 335.38 | 188.2791 | < 2.2e-16 | 88.72501 | 82.79412 |
|  | Protein | 18.05 | 10.1346 | 0.002049 |  |  |
|  | Flour Moisture | 63.15 | 35.4532 | 6.04E-08 |  |  |
|  | AX:BG | 12.58 | 7.0647 | 0.00943 |  |  |
|  | Year | 80.49 | 45.1858 | 2.11E-09 |  |  |
| fibre 12 | Starch damage | 316.063 | 163.8003 | < 2.2e-16 | 87.78649 | 85.12508 |
|  | Protein | 20.989 | 10.8776 | 0.001435 |  |  |
|  | Flour Moisture | 52.817 | 27.3726 | 1.24E-06 |  |  |
|  | WE-G | 0.278 | 0.1439 | 0.705408 |  |  |
|  | Year | 37.249 | 19.3042 | 3.27E-05 |  |  |
| fibre 13 | Starch damage | 319.63 | 165.362 | < 2.2e-16 | 87.76533 | 85.31811 |
|  | Protein | 20.38 | 10.544 | 0.001683 |  |  |
|  | Flour Moisture | 55.2 | 28.557 | 7.83E-07 |  |  |
|  | TOT-G | 0 | 0 | 0.994854 |  |  |
|  | Year | 34.41 | 17.802 | 6.23E-05 |  |  |
| fibre 14 | Starch damage | 212.52 | 110.4944 | < 2.2e-16 | 87.82578 | 84.84845 |
|  | Protein | 21.04 | 10.939 | 0.001393 |  |  |
|  | Flour Moisture | 51.122 | 26.5795 | 1.69E-06 |  |  |
|  | TOT-AX (-X1) | 0.793 | 0.4122 | 0.522622 |  |  |
|  | Year | 41.435 | 21.5431 | 1.28E-05 |  |  |
| fibre 15 | Starch damage | 331.49 | 180.2889 | < 2.2e-16 | 88.36205 | 84.59775 |
|  | Protein | 26.87 | 14.6148 | 0.000254 |  |  |
|  | Flour Moisture | 56.56 | 30.7605 | 3.39E-07 |  |  |
|  | WE-A | 7.82 | 4.2558 | 0.042242 |  |  |
|  | Year | 51.79 | 28.1698 | 9.09E-07 |  |  |
| fibre all PC | Starch damage | 144.03 | 89.3002 | 2.36E-14 | 89.79107 | 89.88465 |
|  | Protein | 20.636 | 12.7948 | 0.000617 |  |  |
|  | Flour Moisture | 32.506 | 20.1539 | 2.58E-05 |  |  |
|  | fibre_PC_1 | 3.7 | 2.2941 | 0.134128 |  |  |
|  | fibre_PC_2 | 6.01 | 3.7266 | 0.057386 |  |  |
|  | fibre_PC_3 | 2.766 | 1.7148 | 0.194417 |  |  |
|  | fibre_PC_4 | 9.678 | 6.0003 | 0.01667 |  |  |
|  | fibre_PC_5 | 7.77 | 4.8177 | 0.031306 |  |  |
|  | fibre_PC_6 | 5.025 | 3.1153 | 0.081686 |  |  |
|  | fibre_PC_7 | 0.457 | 0.2836 | 0.59593 |  |  |
|  | fibre_PC_8 | 0.075 | 0.0462 | 0.830361 |  |  |
|  | fibre_PC_9 | 2.476 | 1.5352 | 0.219249 |  |  |
|  | fibre_PC_10 | 0.069 | 0.0428 | 0.836698 |  |  |
|  | Year | 0.504 | 0.3126 | 0.577807 |  |  |
| fibre reduced PC | Starch damage | 196.428 | 125.1363 | < 2.2e-16 | 90.06424 | 90.18445 |
|  | Protein | 27.278 | 17.3779 | 7.87E-05 |  |  |
|  | Flour Moisture | 38.28 | 24.3869 | 4.39E-06 |  |  |
|  | fibre_PC_1 | 18.742 | 11.94 | 0.000892 |  |  |
|  | fibre_PC_2 | 19.373 | 12.3415 | 0.00074 |  |  |
|  | fibre_PC_4 | 14.978 | 9.542 | 0.002782 |  |  |
|  | fibre_PC_5 | 14.419 | 9.1855 | 0.003309 |  |  |
|  | fibre_PC_6 | 6.052 | 3.8553 | 0.053154 |  |  |
|  | fibre_PC_9 | 4.955 | 3.1567 | 0.079513 |  |  |
|  | Year | 0.069 | 0.0442 | 0.833949 |  |  |

**Table S6.** Contributions (loadings) of fibre components to PCs 1 to 7

| **Percentage variance of each PC** | 39.85 | 23.25 | 17.84 | 8.327 | 4.689 | 2.949 | 2.103 |
| --- | --- | --- | --- | --- | --- | --- | --- |
|  |  |  |  |  |  |  |  |
|  | **PC1** | **PC2** | **PC3** | **PC4** | **PC5** | **PC6** | **PC7** |
| **RV** | -0.27701 | 0.195765 | -0.4036 | -8.05E-02 | 0.221264 | -0.30017 | -0.29182 |
| **WE-X** | -0.27051 | 0.170304 | -0.4461 | 8.75E-02 | 0.052067 | 0.298248 | 0.293284 |
| **WE-A:X** | -0.33217 | 0.154699 | 0.21825 | -2.59E-01 | 0.459094 | -0.05578 | 0.307611 |
| **Sucrose SRC** | -0.34255 | -0.22778 | 0.162774 | 3.48E-01 | -0.10564 | -0.17016 | -0.11571 |
| **TOT-A** | -0.14917 | -0.50715 | -0.16238 | -7.73E-05 | 0.101421 | 0.309097 | 0.107519 |
| **TOT-A:X** | -0.17066 | -0.19424 | 0.489752 | -3.20E-01 | 0.151321 | 0.282399 | 0.212037 |
| **TOT-X** | -0.09184 | -0.45523 | -0.34936 | 1.27E-01 | 0.114523 | 0.210895 | 0.050373 |
| **WE:TOT X** | -0.21507 | 0.456517 | -0.19843 | -3.32E-02 | -0.02579 | 0.166766 | 0.284881 |
| **TOT-BG** | 0.364751 | -0.0357 | -0.08171 | 4.55E-01 | 0.135319 | 0.038297 | 0.373851 |
| **G3:G4 BG** | 0.339636 | 0.219304 | -0.02194 | -1.35E-01 | 0.166953 | 0.642372 | -0.43588 |
| **AX:BG** | -0.39712 | -0.13254 | -0.08008 | -1.54E-01 | -0.21813 | 0.149513 | -0.38871 |
| **WE-G** | 0.202048 | -0.14344 | -0.26251 | -5.82E-01 | -0.51539 | -0.1105 | 0.301818 |
| **TOT-G** | 0.263478 | -0.24243 | -0.23202 | -3.01E-01 | 0.565726 | -0.30435 | -0.093 |

**Table S7.** Regression analysis of WA, adding fibre components to the Baseline equation

| **Model** | **Term** | **F value** | **P value** | **Adjusted R^2^**  **(reported as % variance accounted for)** |
| --- | --- | --- | --- | --- |
| Baseline | Starch damage | 73.5603 | 3.35E-06 | 86.01 |
|  | Protein | 15.5104 | 0.002319 |  |
|  | Flour Moisture | 0.0023 | 0.962908 |  |
| fibre 1 | Starch damage | 177.7883 | 1.08E-07 | 94.21 |
|  | Protein | 37.487 | 0.000112 |  |
|  | Flour Moisture | 0.0055 | 0.942499 |  |
|  | RV | 16.586 | 0.002241 |  |
| fibre 2 | Starch damage | 314.8097 | 6.90E-09 | 96.73 |
|  | Protein | 66.3783 | 1.00E-05 |  |
|  | Flour Moisture | 0.0097 | 0.923544 |  |
|  | WE-(A)X | 37.0758 | 0.000117 |  |
| fibre 3 | Starch damage | 94.3777 | 2.07E-06 | 89.10 |
|  | Protein | 19.8998 | 0.001214 |  |
|  | Flour Moisture | 0.0029 | 0.958086 |  |
|  | TOT-A:X | 4.113 | 0.070033 |  |
| fibre 4 | Starch damage | 83.0704 | 3.69E-06 | 87.61 |
|  | Protein | 17.5156 | 0.001872 |  |
|  | Flour Moisture | 0.0026 | 0.960674 |  |
|  | TOT-(A)X | 2.4221 | 0.150689 |  |
| fibre 5 | Starch damage | 109.9273 | 1.03E-06 | 90.64 |
|  | Protein | 23.1784 | 0.000708 |  |
|  | Flour Moisture | 0.0034 | 0.954769 |  |
|  | WE:TOT(A)X | 6.4382 | 0.029496 |  |
| fibre 6 | Starch damage | 118.6462 | 7.22E-07 | 91.33 |
|  | Protein | 25.0168 | 0.000536 |  |
|  | Flour Moisture | 0.0037 | 0.953011 |  |
|  | TOT-BG | 7.742 | 0.019369 |  |
| fibre all PC | Starch damage | 213.4555 | 0.000128 | 95.18 |
|  | Protein | 45.0076 | 0.00257 |  |
|  | Flour Moisture | 0.0066 | 0.939302 |  |
|  | fibre_PC_1 | 9.3709 | 0.037615 |  |
|  | fibre_PC_2 | 0.5014 | 0.517985 |  |
|  | fibre_PC_3 | 16.1098 | 0.015948 |  |
|  | fibre_PC_4 | 0.1551 | 0.713826 |  |
|  | fibre_PC_5 | 1.2991 | 0.317999 |  |
|  | fibre_PC_6 | 0.4796 | 0.526725 |  |
|  | fibre_PC_7 | 0.0038 | 0.953751 |  |
| fibre PC1 + 3 | Starch damage | 318.2076 | 2.484e-08 | 96.77 |
|  | Protein | 67.0948 | 1.832e-05 |  |
|  | Flour Moisture | 0.0098 | 0.9233477 |  |
|  | fibre_PC_1 | 13.9696 | 0.0046427 |  |
|  | fibre_PC_3 | 24.6142 | 0.0007792 |  |

**Figure S1.** Heat map of correlations between grain parameters.

**Figure S2.** Heat map of correlations between parameters relating to fibre amount and composition.
